# Nutritional status of iron and vitamin D in postpartum women in northern Taiwan

**DOI:** 10.3389/fnut.2025.1701550

**Published:** 2026-01-12

**Authors:** Sing-Chung Li, Yi-Ling Chen, Chiao-Ming Chen, Li-Yi Tsai, Shu-Chi Mu

**Affiliations:** 1 School of Nutrition and Health Sciences, College of Nutrition, Taipei Medical University, Taipei, Taiwan; 2 Department of Pediatrics, Shin-Kong Wu Ho-Su Memorial Hospital, Taipei, Taiwan; 3 Department of Food Science, Nutrition, and Nutraceutical Biotechnology, Shih Chien University, Taipei, Taiwan; 4 School of Medicine, College of Medicine, Fu-Jen Catholic University, New Taipei City, Taiwan

**Keywords:** iron deficiency, iron deficiency anemia, maternal nutrition, postpartum women, supplementation, vitamin D deficiency

## Abstract

**Background:**

Postpartum women are at increased risk of iron and vitamin D deficiencies due to physiological demands, blood loss during delivery, and limited sun exposure during the confinement period. The aimed of this study was to assess the iron and vitamin D nutritional status of women at six weeks postpartum in northern Taiwan and to examine its associations with dietary intake and nutritional supplement use.

**Methods:**

This cross-sectional study included 120 women at six weeks postpartum in northern Taiwan. Dietary intake was assessed using 24-h dietary recall, and supplement use was recorded. Hematological and biochemical parameters, including hemoglobin, ferritin, and serum 25(OH)D_3_, were measured.

**Results:**

The prevalence of iron deficiency and iron deficiency anemia was 5.0% and 6.7%, respectively, while 75% of women exhibited vitamin D insufficiency or deficiency. Supplement users had a significantly lower prevalence of iron deficiency and vitamin D deficiency compared with non-users.

**Conclusion:**

Our findings suggest that dietary intake alone is insufficient to meet postpartum micronutrient requirements and that supplementation plays a critical role in maintaining adequate nutritional status. These results highlight the need for targeted nutritional strategies and updated dietary recommendations to support maternal recovery and health after childbirth.

## 1 Introduction

Postpartum anemia and micronutrient deficiencies remained major public health concerns worldwide. During pregnancy, maternal iron requirements increased substantially to support the expansion of blood volume, fetal growth, and placental development (1). Without adequate dietary intake or supplementation, many women entered the postpartum period with depleted iron stores. Furthermore, blood loss during delivery exacerbated this condition, leading to iron deficiency (ID) and iron deficiency anemia (IDA), both of which were associated with maternal fatigue, impaired cognitive function, reduced quality of life, and negative effects on infant care and development (2, 3).

In addition to iron, vitamin D has emerged as another critical nutrient for maternal and infant health. Vitamin D played an essential role in calcium and bone metabolism, immune function, and muscle strength (4, 5). However, vitamin D deficiency was prevalent among women of reproductive age, particularly in regions with limited sunlight exposure or low dietary intake (6). Recent studies reported high rates of vitamin D insufficiency and deficiency in postpartum women, raising concerns about its impact not only on maternal skeletal health but also on lactational transfer and infant growth (7).

Although iron and vitamin D deficiencies were studied extensively in pregnant populations, data focusing on postpartum women remained relatively limited, especially during the early postpartum period when nutritional demands were high and physiological recovery was critical. Moreover, the role of nutritional supplementation in mitigating these deficiencies had not been fully elucidated in this population (8) Therefore, this study aimed to investigate the prevalence of iron deficiency, iron deficiency anemia, and vitamin D insufficiency among women at six weeks postpartum. In addition, dietary intake, hematological and biochemical markers, and the impact of supplement use on iron and vitamin D nutritional status were evaluated. Findings from this study provided evidence for targeted nutritional strategies to improve maternal health during the postpartum period.

## 2 Materials and methods

### 2.1 Study design and participants

This study was conducted at the Department of Obstetrics and Gynecology, Shin-Kong Wu Ho-Su Memorial Hospital, between January 2016 and September 2017, as part of a project investigating the relationship between postpartum nutritional status and postpartum depression. Women attending routine postpartum follow-up visits were screened for eligibility. The inclusion criteria were women aged 20–50 years at 6–8 weeks postpartum who were in good general health without chronic diseases, were non-smokers and non-drinkers, and had a full-term infant without congenital or medical conditions. The exclusion criteria included: congenital disorders (such as thalassemia, thyroid dysfunction, or diabetes), multiple pregnancies, preterm delivery, individuals who reported acute infectious conditions (e.g., common cold or mastitis) or had total white blood cells (WBCs) counts above or below the normal reference range, or a history of psychiatric disorders (including schizophrenia, depression, or bipolar disorder). A total of 120 women with complete dietary records and biochemical data were included in the secondary analysis of this study. This study was approved by the Institutional Review Board of Shin-Kong Wu Ho-Su Memorial Hospital (approval no. 20150810R). Written informed consent was obtained from all participants prior to enrollment.

### 2.2 Laboratory measurements

Venous blood samples were collected after an overnight fast at 6 weeks postpartum. Hematological parameters including hemoglobin (Hb), hematocrit (HCT), red blood cell count (RBC), and mean corpuscular volume (MCV) were analyzed using an automated hematology analyzer (XN-10, Sysmex, Japan). Serum 25-hydroxyvitamin D_3_ [25(OH)D_3_], parathyroid hormone (PTH), and ferritin were measured by electro-chemiluminescence immunoassay (ECLIA) using a Roche Cobas 6000 analyzer, with inter-assay coefficients of variation (CVs) of 8.64, 3.97, and 2.25%, respectively. Serum calcium was measured using the o-cresolphthalein complexone (oCPC) method with a Beckman Coulter reagent kit, and absorbance was read on a Roche Cobas 8000 analyzer (inter-assay CV = 1.17%). Serum iron and total iron-binding capacity (TIBC) were determined by the Ferrozine colorimetric method on a Roche Cobas 8000 analyzer, with inter-assay CVs of 1.99% and 3.35%, respectively. Transferrin saturation (TS) was calculated by dividing serum iron by total iron-binding capacity (TIBC). Iron nutritional status was classified as follows: normal (ferritin > 15 μg/L and Hb > 12 g/dL), iron deficiency (ID) (ferritin < 15 μg/L), and iron deficiency anemia (IDA) (ferritin < 15 μg/L with Hb < 12 g/dL). Vitamin D nutritional status was categorized as: sufficient [25(OH)D_3_ ≥ 30 ng/mL], insufficient (20–29 ng/mL), and deficient (< 20 ng/mL).

### 2.3 Data collection and dietary assessment

Maternal characteristics were obtained through a structured questionnaire, including anthropometric data (height, pre-pregnancy weight, gestational weight gain, and current weight), as well as sociodemographic information (education level, employment status, parity, and mode of delivery).

Dietary assessment was conducted by a registered dietitian using the 24-h dietary recall method with the aid of food models to improve accuracy. The dietary records reflected the participants’ intake on the previous day. Reported food portions were converted into food weights and analyzed for nutrient composition using the Taiwanese Food Composition Database. For women reporting supplement use during the postpartum period, information on supplement type, frequency, and dosage was collected. Intakes were then converted to standardized daily nutrient values for analysis.

### 2.4 Statistical analysis

All statistical analyses were performed using SPSS software, version 22.0 (IBM Corp., Armonk, NY, USA). The Kolmogorov–Smirnov test was applied to assess the normality of data distribution. Continuous variables were expressed as mean ± standard deviation (SD). For group comparisons, one-way analysis of variance (ANOVA) was performed, followed by Bonferroni *post-hoc* tests, as previously described in studies (9). A two-tailed *p*-value < 0.05 was considered statistically significant. Categorical variables were presented as frequencies and percentages [*n* (%)], and differences between groups were assessed using the chi-square test. Correlations between variables were examined using Pearson correlation analysis. A two-tailed *P*-value < 0.05 was considered statistically significant.

## 3 Results

### 3.1 Participant characteristics at 6 weeks postpartum

At six weeks postpartum, the mean age of participants was 32.4 ± 4.5 years. The average pre-pregnancy BMI was 21.6 ± 3.2 kg/m^2^, with a mean gestational weight gain of 12.9 ± 4.3 kg. At the time of follow-up, the average BMI had increased to 23.4 ± 3.5 kg/m^2^. Regarding education, 80% of the women had completed ≥ 16 years of education, while 20% had ≤ 12 years. In terms of working status, nearly half of the participants were employed full-time (48.3%), while 35.0% were homemakers and 15.8% worked part-time, with one case missing data. A majority were primiparas (62.5%), and the remainder multiparas (37.5%). The mode of delivery was predominantly vaginal (66.7%) compared with 33.3% who delivered via cesarean section. Most participants reported taking nutritional supplements during pregnancy, particularly in the first (92.5%) and second (84.2%) trimesters, with slightly lower rates in the third trimester (80.0%). During lactation, however, the proportion of supplement use decreased to 51.7% (Table 1).

**TABLE 1:** Characteristic of women 6 weeks postpartum.

Variables	Total subjects (*n* = 120)
Age (year)	32.4 ± 4.5
**Anthropometric**	
Before pregnancy BMI (kg/m^2^)	21.6 ± 3.2
Gestational weight gain (kg)	12.9 ± 4.3
Currently BMI (kg/m^2^)	23.4 ± 3.5
**Education years**	
≤ 12	24 (20)
≥ 16	96 (80)
**Working state**	
Homemaking	42 (35.0)
Full-time	58 (48.3)
Part-time	19 (15.8)
**Parity**	
Primipara	75 (62.5)
Multipara	45 (37.5)
**Type of delivery**	
Vaginal	80 (66.7)
Caesarian section	40 (33.3)
**Nutritional supplements**	
Pregnancy first trimester	111 (92.5)
Pregnancy second trimester	101 (84.2)
Pregnancy third trimester	96 (80.0)
Lactation	62 (51.7)

Values are presented as mean ± SD or number (percentage).

### 3.2 Nutrients intake and iron nutritional status of 6 weeks postpartum women

Iron nutritional status was classified as follows: Normal, ferritin > 15 μg/L and hemoglobin > 12 g/dL; Iron deficiency (ID), ferritin < 15 μg/L; Iron deficiency anemia (IDA), ferritin < 15 μg/L and hemoglobin < 12 g/dL. Based on these criteria, among the 120 women at six weeks postpartum, 8 participants (6.7%) were identified with IDA, while 6 participants (5.0%) had ID, and the remaining 106 (88.3%) had normal iron status (Table 2).

**TABLE 2:** Nutrients intake and iron nutritional status of 6 weeks postpartum women.

Variables	Normal (*n* = 106)	ID (*n* = 6)	IDA (*n* = 8)	*P*-value
**Nutrients intake**				
Calories (Kcal)	1,513.6 ± 481.2	1,324.0 ± 343.9	1,313.2 ± 464.4	0.353
Carbohydrates (g)	189.0 ± 72.3	176.7 ± 59.4	162.6 ± 71.5	0.570
Fat (g)	55.6 ± 24.7	44.8 ± 11.3	50.0 ± 20.6	0.476
Protein (g)	65.2 ± 24.3	57.3 ± 13.1	54.4 ± 17.2	0.356
Iron (mg)	9.5 ± 10.2	10.4 ± 7.1	8.0 ± 4.1	0.893
Total iron intake (mg)	19.8 ± 15.6	19.1 ± 14.2	9.2 ± 7.1	0.163
**Iron nutritional status**				
HB (g/dL)	13.3 ± 1.0^a^	12.5 ± 0.3^ab^	10.8 ± 0.7^b^	< 0.001
RBC (106/uL)	4.6 ± 0.4	4.7 ± 0.3	4.4 ± 0.4	0.183
MCV (fL)	87.3 ± 6.0^a^	82.9 ± 4.1^ab^	79.7 ± 8.0^b^	0.004
HCT (%)	40.2 ± 2.7^a^	39.1 ± 0.9^ab^	34.9 ± 1.7^b^	< 0.001
Ferritin (μg/L)	64.3 ± 51.0^a^	11.0 ± 2.8^b^	9.0 ± 2.3^b^	0.001
TS (%)	25.9 ± 11.9^a^	16.7 ± 4.2^ab^	7.8 ± 1.0^b^	< 0.001

Values are presented as mean ± SD. Statistical significance was assessed using one-way ANOVA followed by Bonferroni *post-hoc* tests. A two-tailed *p*-value < 0.05 was considered statistically significant. Different superscript letters indicate significant differences between groups. ID, iron deficiency; IDA, iron deficiency anemia; HB, hemoglobin; RBC, red blood cell; MCV, mean corpuscular volume; HCT, hematocrit; TS, transferrin saturation. Iron nutritional status was classified as follows: Normal, ferritin > 15 μg/L and hemoglobin > 12 g/dL; Iron deficiency, ferritin < 15 μg/L; Iron deficiency anemia, ferritin < 15 μg/L, hemoglobin < 12 g/dL. Total iron intake included dietary intake and supplements.

No significant differences were found in energy and macronutrient intakes across the three groups (*P* > 0.05). However, significant variations were observed in hematological and biochemical markers. Women with IDA exhibited substantially lower hemoglobin (10.8 ± 0.7 g/dL) compared with both normal (13.3 ± 1.0 g/dL) and ID (12.5 ± 0.3 g/dL) groups (*P* < 0.001). Hematocrit and mean corpuscular volume were also significantly reduced in the IDA group (34.9 ± 1.7% and 79.7 ± 8.0 fL, respectively; *P* < 0.01). Furthermore, serum ferritin and transferrin saturation decreased markedly with worsening iron status, being lowest in IDA (9.0 ± 2.3 μg/L and 7.8 ± 1.0%) compared with ID (11.0 ± 2.8 μg/L and 16.7 ± 4.2%) and normal women (64.3 ± 51.0 μg/L and 25.9 ± 11.9%; *P* ≤ 0.001).

### 3.3 Nutrient intake and vitamin D status at 6 weeks postpartum

Vitamin D status was classified as follows: sufficient [serum 25(OH)D_3_ ≥ 30 ng/mL], insufficient (20–29 ng/mL), and deficient (< 20 ng/mL). Based on these criteria, 38.3% of participants were vitamin D deficient, while an additional 37.5% were insufficient, indicating a high prevalence of suboptimal vitamin D status among women at six weeks postpartum (Table 3).

**TABLE 3:** Nutrients intake and vitamin D nutritional status of 6 weeks postpartum women.

Variables	Sufficient (*n* = 29)	Insufficient (*n* = 45)	Deficiency (*n* = 46)	*P*-value
**Nutrients intake**				
Calories (Kcal)	1,429 ± 461	1,501 ± 486	1,520 ± 480	0.712
Carbohydrates (g)	165.7 ± 68.3	191.9 ± 77.8	194.6 ± 65.7	0.193
Fat (g)	57.2 ± 23.3	53.1 ± 24.7	54.8 ± 24.1	0.771
Protein (g)	64.6 ± 26.5	64.6 ± 21.1	63.2 ± 24.4	0.952
Vitamin D (μg)	5.2 ± 8.2	5.7 ± 7.3	5.7 ± 6.7	0.957
Total vitamin D intake (μg)	15.1 ± 12.9^a^	11.3 ± 9.4^ab^	8.1 ± 8.5^b^	0.014
**Vitamin D nutritional status**				
Serum 25(OH)D_3_	35.3 ± 4.0^a^	25.4 ± 3.0^b^	16.6 ± 2.6^c^	< 0.001
Serum Ca (mg/dL)	9.7 ± 0.6^a^	9.5 ± 0.4^ab^	9.3 ± 0.4^b^	0.005
Serum PTH (pg/mL)	31.0 ± 15.8	35.6 ± 15.7	38.5 ± 14.7	0.119

Values are presented as mean ± SD. Statistical significance was assessed using one-way ANOVA followed by Bonferroni *post-hoc* tests. A two-tailed *p*-value < 0.05 was considered statistically significant. Different superscript letters indicate significant differences between groups. Vitamin D status was classified as sufficient (≥ 30 ng/mL), insufficient (20–29 ng/mL), or deficient (< 20 ng/mL). PTH, parathyroid hormone, total vitamin D intake included dietary intake and supplements.

Dietary energy and macronutrient intakes did not differ significantly across vitamin D status groups (*P* > 0.05). However, vitamin D intake showed a significant gradient, with the highest intake observed in the sufficient group (15.1 ± 12.9 μg/day) compared to the deficient group (8.1 ± 8.5 μg/day, *P* = 0.014). Serum biomarkers further confirmed these differences. Women with sufficient vitamin D had the highest serum 25(OH)D_3_ concentrations (35.3 ± 4.0 ng/mL), which were significantly greater than those in the insufficient (25.4 ± 3.0 ng/mL) and deficient (16.6 ± 2.6 ng/mL) groups (*P* < 0.001). Serum calcium levels also showed a modest but significant decline with worsening vitamin D status (from 9.7 ± 0.6 mg/dL in the sufficient group to 9.3 ± 0.4 mg/dL in the deficient group, *P* = 0.005). Although serum PTH levels were higher in deficient women, the difference was not statistically significant (*P* = 0.119).

**FIGURE 1:**
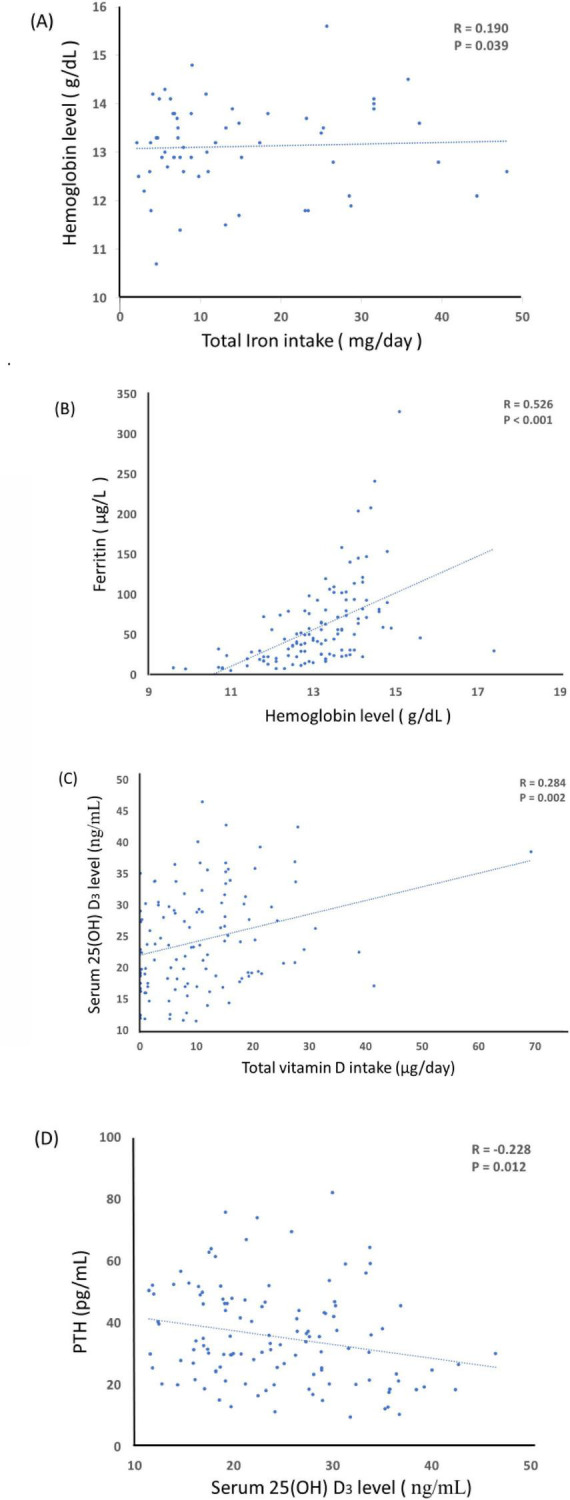
Correlations between nutrient intake and biochemical parameters at 6 weeks postpartum. **(A)** Relationship between total iron intake and hemoglobin level (*r* = 0.190, *P* = 0.039). **(B)** Relationship between hemoglobin level and serum ferritin concentration (*r* = 0.526, *P* < 0.001). **(C)** Relationship between total vitamin D intake and serum 25(OH)D_3_ concentration (*r* = 0.284, *P* = 0.016). **(D)** Relationship between serum 25(OH)D_3_ and serum parathyroid hormone (PTH) concentration (*r* = –0.228, *P* = 0.012).

Correlation analyses between nutrient intake and biochemical markers are presented in Figure 1. A modest but significant positive association was observed between total iron intake and hemoglobin level (*r* = 0.190, *P* = 0.039; Figure 1A). Hemoglobin levels were also strongly correlated with serum ferritin concentrations (*r* = 0.526, *P* < 0.001; Figure 1B). Regarding vitamin D, total vitamin D intake was positively associated with serum 25(OH)D_3_ concentrations (*r* = 0.284, *P* = 0.016; Figure 1C). Conversely, serum 25(OH)D_3_ levels were inversely correlated with serum parathyroid hormone (PTH) (*r* = −0.228, *P* = 0.012; Figure 1D). These findings suggest that higher dietary iron and vitamin D intakes are associated with better biochemical markers of iron and vitamin D status, respectively, while low vitamin D status is linked to elevated PTH levels.

### 3.4 Comparison of iron and vitamin D status with and without supplement use

Iron and vitamin D status were further compared between women who reported using nutritional supplements and those who did not. For iron status, the prevalence of deficiency and anemia was significantly lower in the supplement group (*P* = 0.025). Specifically, 96.6% of women using supplements had normal iron status, compared with 80.6% among non-users. In contrast, iron deficiency (8.1%) and iron deficiency anemia (11.3%) were more frequent in women without supplement use, whereas both conditions were rare among supplement users (1.7% each) (Table 4).

**TABLE 4:** Comparison of iron and vitamin D nutritional status with and without supplement use.

Variables	With supplement *n* (%)	Without supplement *n* (%)	*P*-value
**Iron nutritional status**			0.025
Normal	56 (96.6)	50 (80.6)	
Iron deficiency	1 (1.7)	7 (8.1)	
Iron deficient anemia	1 (1.7)	5 (11.3)	
**Vitamin D nutritional status**			< 0.001
Sufficient	21 (32.8)	8 (14.3)	
Insufficient	29 (45.3)	16 (28.6)	
Deficiency	14 (21.9)	32 (57.1)	

Values are presented as number (percentage). Statistical significance was determined using chi-squared test (*p* < 0.05). Iron nutritional status was classified as follows: Normal, ferritin > 15 μg/L and hemoglobin > 12 g/dL; Iron deficiency, ferritin < 15 μg/L; Iron deficiency anemia, ferritin < 15 μg/L, hemoglobin < 12 g/dL. Vitamin D status was classified as sufficient (≥ 30 ng/mL), insufficient (20–29 ng/mL), or deficient (< 20 ng/mL).

Vitamin D status also differed markedly by supplement use (*P* < 0.001). Among supplement users, 32.8% achieved sufficiency compared with only 14.3% of non-users. Conversely, vitamin D deficiency was far more prevalent in non-users (57.1%) than in supplement users (21.9%). These findings highlight the potential protective role of supplementation in reducing postpartum iron deficiency and vitamin D insufficiency.

## 4 Discussion

In this study, we investigated iron and vitamin D nutritional status among women at six weeks postpartum and examined the influence of dietary intake and supplement use. Several important findings were observed. First, despite no significant differences in energy and macronutrient intakes across groups, iron deficiency (5.0%) and iron deficiency anemia (6.7%) were still present, highlighting the persistence of iron related problems in the early postpartum period. Second, vitamin D insufficiency (37.5%) and deficiency (38.3%) were highly prevalent, indicating that suboptimal vitamin D status is widespread among postpartum women. Third, supplement use was associated with a lower prevalence of both iron and vitamin D deficiencies, suggesting its potential role in improving maternal micronutrient status. Finally, correlation analyses indicated that higher dietary iron intake was positively associated with hemoglobin, and vitamin D intake was positively associated with serum 25(OH)D_3_, suggesting that diet plays a major role in determining iron and vitamin D nutritional status in postpartum women.

Postpartum anemia represents a significant public health concern. It has been associated with adverse maternal outcomes, including feelings of hopelessness, emotional in-stability, fatigue, increased susceptibility to infections, and reduced quality of life (1). Moreover, maternal iron deficiency and anemia have been linked to impaired cognitive and emotional functioning, which may increase the risk of postpartum depression (10, 11). In addition, maternal anemia may compromise caregiving capacity and is associated with insufficient breast milk production, thereby potentially affecting infant care and overall wellbeing (12). Postpartum anemia is a multifactorial condition, and its etiology reflects both acute and chronic influences on maternal iron status. The most immediate cause is blood loss during delivery, with postpartum hemorrhage being a leading contributor to acute declines in hemoglobin levels. In addition, many women enter the postpartum period with pre-existing iron deficiency acquired during pregnancy, due to increased maternal blood volume and fetal demands. Without sufficient dietary intake or supplementation, these depleted iron stores are not adequately restored after birth. Although lactation itself does not substantially increase iron losses, the overall elevated nutritional demands during breastfeeding may exacerbate iron deficiency in women with marginal dietary in-take. Taken together, these factors highlight the complex interplay of obstetric events, maternal nutrition, and health status in shaping postpartum anemia risk (2, 3). In our study, although a relatively high proportion of women delivered by cesarean section (33.3%), no significant association was observed between cesarean delivery and the prevalence of iron deficiency or iron deficiency anemia. This contrasts with previous studies reporting higher rates of postpartum anemia following cesarean section. For example, a study in Ethiopia found that nearly one in five women (18.9%) who underwent cesarean delivery developed postpartum anemia (13), while research from Uganda reported that 6.8% of women experienced severe anemia within three days after cesarean section (14). The discrepancy may be attributed to differences in population characteristics, baseline nutritional status, intraoperative blood management, and access to postpartum care.

According to previous surveys in Taiwan, the prevalence of anemia and iron deficiency among women of reproductive age and pregnant women varies by study population and assessment criteria. Among pregnant women in northern Taiwan (2017–2019), the prevalence of iron deficiency, and iron deficiency anemia (IDA) was 37.7%, and 9.3%, respectively (15). In a nationwide survey of pregnant women at 24–28 weeks of gestation, the prevalence of anemia (defined as Hb < 10.5 g/dL) was 17.4% (16). Collectively, these findings indicate that anemia and iron deficiency remain common among Taiwanese women during pregnancy. In the present study, we found that total iron intake at six weeks postpartum was positively associated with hemoglobin (Hb) concentrations, and Hb levels were further correlated with body iron stores, indicating that adequate iron intake and storage are essential for hemoglobin synthesis and the prevention of anemia. Interestingly, our results showed no significant differences in macronutrient or dietary iron intake among women with normal iron status, iron deficiency, and iron deficiency anemia, suggesting that diet alone may not be sufficient to provide adequate iron to prevent iron deficiency and anemia. Importantly, our data revealed that nearly all cases of iron deficiency and iron deficiency anemia occurred among women who did not take iron supplements. These findings suggest that, in addition to dietary practices during the postpartum period, appropriate use of supplementation may play a more decisive role in preventing iron deficiency anemia.

In Taiwan, postpartum women commonly follow the traditional practice of Zuo Yuezi (the confinement period), during which mothers consume larger amounts of iron-rich protein foods such as pork, beef, eggs, and organ meats. This cultural practice may help replenish iron stores depleted during pregnancy. However, few studies have examined whether this postpartum confinement period sufficiently restores maternal iron status. Therefore, we aimed to assess women’s iron nutritional status at six weeks postpartum, after completing the Zuo Yue Zi period. Nevertheless, this timing may miss the peak of iron deficiency, which typically occurs at 2–4 weeks postpartum. Future studies with earlier screening and targeted dietary management for women with poor iron status may help reduce the prevalence of postpartum iron deficiency.

The importance of iron supplementation during the postpartum period has been confirmed by several randomized controlled trials. In mothers with postpartum depression, early oral iron supplementation (50 mg/day) significantly improved serum ferritin levels and reduced iron deficiency prevalence from 31.4% to 8.5%, accompanied by a 42.8% improvement in depressive symptoms (17). Intravenous iron administration has been shown to be even more effective; postpartum women receiving IV iron demonstrated significantly higher hemoglobin levels at six weeks compared to those on oral iron (18). A universal supplementation approach in low-income postpartum women similarly resulted in significant anemia reduction: Hb increased and anemia prevalence dropped to 22.5% with daily 65 mg iron supplementation for two months (19). Even among lactating women, iron supplementation yielded moderate improvements in iron status, particularly in those without elevated inflammatory markers, highlighting the continued benefit of maintaining iron intake during the postpartum phase (20).

Vitamin D deficiency has emerged as a global public health concern, and pregnant women represent one of the most vulnerable groups. Numerous studies have reported a high prevalence of vitamin D insufficiency and deficiency during pregnancy, particularly in Asian populations. In Taiwan, a prospective study of 125 pregnant women reported a mean serum 25(OH)D_3_ concentration of only 17.2 ng/mL, with more than two-thirds of participants classified as vitamin D deficient or severely deficient (21). These findings are consistent with other regional and international data, which suggest that vitamin D deficiency affects over half of pregnant women worldwide (22). Our results also showed that 75% of women at six weeks postpartum were in a state of vitamin D insufficiency or deficiency, a prevalence comparable to that reported in previous studies during pregnancy. Vitamin D can be synthesized in the skin through sunlight exposure. Several studies have indicated that living in regions with limited sunlight or having reduced outdoor activity lowers vitamin D status (5, 23). A study from Slovenia reported that women with less frequent outdoor activities and those who became pregnant during seasons with lower sunlight exposure had a higher risk of vitamin D deficiency (24). Similarly, a multicenter retrospective cohort study in Jiangsu Province found that less than one hour of outdoor activity per day during lactation was significantly associated with an increased risk of vitamin D deficiency in infants (25). Therefore, we believed that the lack of outdoor activity during the Zuo Yue Zi (postpartum confinement) period may negatively affect maternal vitamin D status. The findings highlight that cultural confinement, combined with limited sun exposure at Taiwan’s latitude, likely contributes to vitamin D deficiency, suggesting that dietary or supplemental vitamin D interventions should be considered in postpartum care.

In our study, dietary vitamin D intake did not differ significantly between women with sufficient and deficient vitamin D status. However, when supplementation was taken into account, women in the sufficient group had a markedly higher total vitamin D intake compared with those in the deficient group. Moreover, serum 25(OH)D_3_ concentrations were positively correlated with total vitamin D intake. In addition, we observed an inverse relationship between serum 25(OH)D_3_ and parathyroid hormone (PTH) concentrations. Vitamin D deficiency impairs active calcium absorption and thereby lowering ionized calcium and stimulating compensatory secretion of PTH (26, 27). Elevated PTH acts to restore serum calcium primarily by increasing bone resorption and phosphate excretion. During lactation, the maternal calcium requirement rises substantially, as 200–300 mg of calcium is secreted daily into human milk. This process is coordinated by mammary-derived parathyroid hormone–related peptide (PTHrP), reduced estrogen levels, and enhanced RANKL signaling, which collectively favor skeletal calcium mobilization (28, 29). When vitamin D is insufficient, the compensatory PTH response becomes exaggerated, leading to accelerated bone turnover and transient loss of bone mineral density, particularly at trabecular-rich sites such as the lumbar spine (30). Although bone mineral content typically recovers after weaning, sustained vitamin D deficiency may impair remineralization and increase the risk of maternal osteopenia or fractures (31). These findings underscore the importance of maintaining adequate vitamin D and calcium intake during lactation to prevent excessive PTH activation and preserve maternal bone health.

Vitamin D supplementation during the postpartum and lactation period provides multiple benefits for mothers. Beyond reducing maternal bone loss, several studies have also demonstrated that vitamin D supplementation may help alleviate postpartum depression. For instance, Rouhi et al. (32) reported that daily supplementation with 1,000 IU of vitamin D for six months significantly improved depressive symptoms among women at high risk of postpartum depression (32). Similarly, Amini et al. (33) found that biweekly supplementation with 50,000 IU of vitamin D_3_ for eight weeks reduced depressive symptoms more effectively than placebo (33). High-dose maternal supplementation (e.g., 6,400 IU/day) has also been shown to safely increase serum 25(OH)D_3_ concentrations and enhance the vitamin D content of breast milk, thereby ensuring adequate vitamin D intake for exclusively breastfed infants without requiring direct infant supplementation (34). However, in our previous study, no association was observed between postpartum depression and maternal vitamin D status (35). This discrepancy may be attributed to differences in supplementation dose, study design, or participant characteristics. A recent systematic review and meta-analysis further supports the complexity of this relationship, reporting that while low maternal 25(OH)D_3_ concentrations are consistently associated with an increased risk of postpartum depression, the evidence from intervention trials remains limited and heterogeneous (36). Taken together, these findings high-light the need for further large-scale, well-controlled randomized trials to clarify the potential role of vitamin D supplementation in preventing or alleviating postpartum depression.

Our results showed that women with sufficient vitamin D status had a mean total vitamin D intake of 15 μg per day, of which approximately 5 μg was obtained from dietary sources. This suggests that postpartum women may require an additional daily supplementation of around 10 μg to maintain adequate vitamin D status. In the current Taiwanese dietary reference intakes (DRIs), the recommended dietary allowance for women of reproductive age is 10 μg/day, with no additional intake specified during lactation. However, our findings indicate that this level of intake may be insufficient for postpartum women to sustain adequate serum vitamin D concentrations. In contrast, the US Institute of Medicine (IOM) recommends a higher intake of 15 μg/day (600 IU) for both reproductive-age and lactating women (37). This discrepancy highlights the possibility that current Taiwanese recommendations may underestimate the actual requirements of postpartum women, particularly considering limited sun exposure during the confinement period and the increased physiological demands of lactation. Further research is therefore warranted to establish evidence-based vitamin D requirements tailored for lactating women in Taiwan.

Although previous studies in Taiwan have reported the prevalence and status of iron and vitamin D deficiencies among pregnant women (15, 38), there is a lack of research investigating this nutritional status in postpartum women. The strength of the present study lies in being one of the few in Taiwan to assess the nutritional status of postpartum women, thereby filling an important gap in this area of research. However, investigations focusing specifically on the iron and vitamin D status of postpartum women are nearly absent. The strength of the present study lies in being one of the few in Taiwan to assess the nutritional status of postpartum women, thereby filling an important research gap in this population. This study has several limitations that should be acknowledged. First, the sample size was relatively small and derived from a single medical center in northern Taiwan, which may limit the generalizability of the findings to other regions or populations with different dietary habits and sunlight exposure. Second, dietary intake was assessed using a single 24-h dietary recall, which may not fully capture habitual intake and is subject to recall bias. Third, although supplement use was recorded, detailed information on compliance, duration, and brand formulation was limited, potentially affecting the accuracy of estimated nutrient intake. Fourth, serum 25(OH)D_3_ and ferritin levels were measured only once at six weeks postpartum, providing a cross-sectional snapshot rather than longitudinal changes throughout the postpartum period. Fifth, potential confounders such as seasonal variation in sunlight exposure, physical activity, and other micronutrient status were not fully controlled, which may have influenced vitamin D and iron status. Finally, the study did not directly assess clinical outcomes such as maternal functional status, bone health, or infant growth, which could further elucidate the implications of micronutrient deficiencies. Future large-scale, multicenter, and longitudinal studies are needed to confirm these findings and establish evidence-based nutritional recommendations for postpartum women. Nevertheless, these findings hold significant implications for enhancing postpartum nutrition and health policy in Taiwan. Given the high prevalence of iron and vitamin D insufficiency among postpartum women, incorporating routine nutritional assessments into postpartum follow-up visits could be beneficial within national maternal health programs. Moreover, integrating evidence-based dietary guidance into traditional Zuo Yue Zi (postpartum confinement) practices—such as encouraging balanced consumption of iron-rich and vitamin D–enhancing foods, appropriate supplementation, and safe sunlight exposure—may help restore maternal micronutrient reserves. In addition, strengthening postpartum nutrition education through the recommendations of the National Health Promotion Administration could aid in preventing long-term nutrient deficiencies and in supporting the overall health and wellbeing of both mothers and their infants.

## 5 Conclusion

In this study, we examined the iron and vitamin D nutritional status of women at six weeks postpartum. Our findings revealed that 5.0% of participants were iron deficient and 6.7% had iron deficiency anemia, whereas as many as 75% were classified as vitamin D insufficient or deficient. Dietary intake of iron and vitamin D alone was not sufficient to distinguish between women with normal and deficient status, highlighting the limitations of diet in meeting increased postpartum micronutrient requirements. Importantly, women who reported supplement use had significantly lower prevalence of iron deficiency, iron deficiency anemia, and vitamin D deficiency compared with those who did not. These results suggest that appropriate supplementation plays a critical role in maintaining adequate micronutrient status during the postpartum period. Taken together, our findings underscore the high prevalence of vitamin D deficiency and the continuing risk of iron deficiency anemia among postpartum women, and emphasize the need for targeted nutritional interventions and updated dietary recommendations to support maternal health and recovery after childbirth.

## Data Availability

The original contributions presented in this study are included in this article/supplementary material, further inquiries can be directed to the corresponding author.

## References

[1] Milman N. Postpartum Anemia I: definition, prevalence, causes, and consequences. *Ann Hematol.* (2011) 90:1247–53. 10.1007/s00277-011-1279-z 21710167

[2] Milman N. Postpartum Anemia II: prevention and treatment. *Ann Hematol.* (2012) 91:143–54. 10.1007/s00277-012-1435-3 22160256

[3] Pavord S Daru J Prasannan N Robinson S Stanworth S Girling J. UK guidelines on the management of iron deficiency in pregnancy. *Br J Haematol.* (2020) 188:819–30. 10.1111/bjh.16221 31578718

[4] Holick MF. Vitamin D deficiency. *New Engl J Med.* (2007) 357:266–81. 10.1056/NEJMra070553 17634462

[5] Wu S-E Chen W-L. Moderate sun exposure is the complementor in insufficient Vitamin D consumers. *Front Nutr.* (2022) 9:832659. 10.3389/fnut.2022.832659 35350415 PMC8957913

[6] Aghajafari F Nagulesapillai T Ronksley PE Tough SC O’Beirne M Rabi DM. Association between maternal serum 25-Hydroxyvitamin D level and pregnancy and neonatal outcomes: systematic review and meta-analysis. *BMJ.* (2013) 346:f1169. 10.1136/bmj.f1169 23533188

[7] Elsori DH Hammoud MS. Vitamin D deficiency in mothers, neonates and children. *J Steroid Biochem Mol Biol.* (2018) 175:195–9. 10.1016/j.jsbmb.2017.01.023 28179126

[8] World Health Organization [WHO]. *Who Recommendations on Antenatal Care for a Positive Pregnancy Experience.* Geneva: World Health Organization (2016). 28079998

[9] Beraki GG Tesfamariam EH Gebremichael A Yohannes B Haile K Tewelde S Knowledge on postnatal care among postpartum mothers during discharge in maternity hospitals in asmara: a cross-sectional study. *BMC Pregnancy Childbirth.* (2020) 20:17. 10.1186/s12884-019-2694-8 31906883 PMC6945610

[10] Corwin EJ Murray-Kolb LE Beard JL. Low hemoglobin level is a risk factor for postpartum depression. *J Nutr.* (2003) 133:4139–42. 10.1093/jn/133.12.4139 14652362

[11] Beard JL Hendricks MK Perez EM Murray-Kolb LE Berg A Vernon-Feagans L Maternal iron deficiency anemia affects postpartum emotions and cognition. *J Nutr.* (2005) 135:267–72. 10.1093/jn/135.2.267 15671224

[12] Beard JL. Why iron deficiency is important in infant development. *J Nutr.* (2008) 138:2534–6. 10.3945/jn.108.098970 19022985 PMC3415871

[13] Habtamu G Talie A Kassa T Belay DM. Prevalence and associated factors of postpartum anemia after cesarean delivery in public hospitals of Awi Zone, North West ethiopia, 2023; a cross-sectional study. *PLoS One.* (2025) 20:e0311907. 10.1371/journal.pone.0311907 39854312 PMC11760021

[14] Sivahikyako SA Owaraganise A Tibaijuka L Agaba DC Kayondo M Ngonzi J Prevalence and factors associated with severe anaemia post-caesarean section at mbarara regional referral hospital, Uganda. *BMC Pregnancy Childbirth.* (2021) 21:674. 10.1186/s12884-021-04157-x 34610802 PMC8493736

[15] Cheng N-H Chao JC-J Bai C-H Chen Y-C Huang Y-L Wang F-F Factors associated with iron status and gestational anemia among pregnant women in northern Taiwan. *J Nutr Sci.* (2022) 46:76–89. 10.6691/NSJ.202209_46(3).0001

[16] Health Promotion Administration, Ministry of Health Welfare Taiwan. *Nutrition and Health Survey in Taiwan (Nahsit): Abnormal Anemia Rate among Pregnant Women at 24–28 Weeks.* Taiwan: HPA (2019).

[17] Sheikh M Hantoushzadeh S Shariat M Farahani Z Ebrahiminasab O. The efficacy of early iron supplementation on postpartum depression. *Eur J Nutr.* (2017) 56:901–8. 10.1007/s00394-015-1140-6 26715522

[18] Saad AF Stepanek R Kothmann M Wilson-Jimenez M McCoy L Aguillon B Intravenous iron compared with oral iron supplementation for postpartum anemia. *Obstet Gynecol.* (2023) 141:1052–60. 10.1097/AOG.0000000000005143 37486650

[19] Mitra AK Khoury AJ. Universal iron supplementation: a simple and effective strategy to reduce anemia among low-income postpartum women. *Public Health Nutrition.* (2012) 15:546–53. 10.1017/S1368980011001261 21729466

[20] Jorgensen JM Yang Z Lönnerdal B Chantry CJ Dewey KG. Effect of iron supplementation during lactation on maternal iron status and oxidative stress: a randomized controlled trial. *Mater Child Nutr.* (2017) 13:e12394. 10.1111/mcn.12394 27896921 PMC6866113

[21] Lin CH Lin PS Lee MS Lin CY Sung YH Li ST Associations between Vitamin D deficiency and carbohydrate intake and dietary factors in Taiwanese pregnant women. *Medicina* (2023) 59:107. 10.3390/medicina59010107 36676731 PMC9863845

[22] Saraf R Morton SM Camargo CA Jr. Grant CC. Global summary of maternal and newborn Vitamin D status - a systematic review. *Maternal Child Nutr.* (2016) 12:647–68. 10.1111/mcn.12210 26373311 PMC6860156

[23] Le Goaziou MF Souberbielle JC Dupraz C Martin A Laville M Schott-Pethelaz AM Risk factors for vitamin d deficiency in women aged 20–50: an observational study. *Eur J General Practi.* (2011) 17:146–52. 10.3109/13814788.2011.560663 21348788

[24] Soltirovska Salamon A Benedik E Bratanič B Velkavrh M Rogelj I Fidler Mis N Vitamin D status and its determinants in healthy slovenian pregnant women. *Ann Nutr Metab.* (2015) 67:96–103. 10.1159/000439093 26340437

[25] Ye K Chang W Zheng W Ni Y Jiang X Wu A Association of Chinese maternal lifestyle during pregnancy and lactation with Vitamin D levels in offspring: a multicenter retrospective cohort jiangsu study. *Food Funct.* (2025) 16:6622–36. 10.1039/d5fo01812a 40709502

[26] Heaney RP Dowell MS Hale CA Bendich A. Calcium absorption varies within the reference range for serum 25-Hydroxyvitamin D. *J Am Coll Nutr.* (2003) 22:142–6. 10.1080/07315724.2003.10719284 12672710

[27] O’Callaghan KM Funk C Fariha F Nagaria MH Dasiewicz A Harrington J Serum 25-Hydroxyvitamin D and intact parathyroid hormone as functional biomarkers of bone mass in early childhood. *J Nutr.* (2025) 155:1782–94. 10.1016/j.tjnut.2025.03.022 40139482 PMC12264547

[28] Kovacs CS. Maternal mineral and bone metabolism during pregnancy, lactation, and post-weaning recovery. *Physiol Rev.* (2016) 96:449–547. 10.1152/physrev.00027.2015 26887676

[29] Kovacs CS Ralston SH. Presentation and management of osteoporosis in pregnancy and lactation. *Endocrine* (2015) 49:353–64. 10.1007/s12020-014-0501-0 25939309

[30] Chen X Shen L Gao C Weng R Fan Y Xu S Vitamin D status and its associations with bone mineral density, bone turnover markers, and parathyroid hormone in chinese postmenopausal women with osteopenia and osteoporosis. *Front Nutr.* (2023) 10:1307896. 10.3389/fnut.2023.1307896 38268673 PMC10806182

[31] Scioscia MF Zanchetta MB. Recent insights into Pregnancy and Lactation-associated Osteoporosis (PLO). *Int J Womens Health.* (2023) 15:1227–38. 10.2147/ijwh.S366254 37551335 PMC10404404

[32] Rouhi M Rouhi N Mohamadpour S Tajrishi HP-R. Vitamin D reduces postpartum depression and fatigue among Iranian women. *Br J Midwifery.* (2018) 26:787–93. 10.12968/bjom.2018.26.12.787

[33] Amini S Amani R Jafarirad S Cheraghian B Sayyah M Hemmati AA. The effect of Vitamin D and calcium supplementation on inflammatory biomarkers, estradiol levels and severity of symptoms in women with postpartum depression: a randomized double-blind clinical trial. *Nutr Neurosci.* (2022) 25:22–32. 10.1080/1028415x.2019.1707396 31900080

[34] Dawodu A Salameh KM Al-Janahi NS Bener A Elkum N. The effect of high-dose postpartum maternal Vitamin D supplementation alone compared with maternal plus infant Vitamin D supplementation in breastfeeding infants in a high-risk population. a randomized controlled trial. *Nutrients.* (2019) 11:1632. 10.3390/nu11071632 31319554 PMC6682993

[35] Lin YH Chen CM Su HM Mu SC Chang ML Chu PY Association between postpartum nutritional status and postpartum depression symptoms. *Nutrients.* (2019) 11:1204. 10.3390/nu11061204 31141947 PMC6628029

[36] Wang J Liu N Sun W Chen D Zhao J Zhang W. Association between Vitamin D deficiency and antepartum and postpartum depression: a systematic review and meta-analysis of longitudinal studies. *Arch Gynecol Obstet.* (2018) 298:1045–59. 10.1007/s00404-018-4902-6 30264203

[37] Institute of Medicine (US) Committee to Review Dietary Reference Intakes for Vitamin D and Calcium, Ross A Taylor C Yaktine A Del Valle H. *Dietary Reference Intakes for Calcium and Vitamin D.* Washington DC: National Academies Press (2011). 21796828

[38] Li WJ Chen KH Huang LW Tsai YL Seow KM. Low maternal serum 25-hydroxyvitamin D concentration is associated with postpartum hemorrhage: a retrospective observational study. *Front Endocrinol.* (2022) 13:816480. 10.3389/fendo.2022.816480 35370939 PMC8968120

